# Utilizing past and present mouse systems to engineer more relevant pancreatic cancer models

**DOI:** 10.3389/fphys.2014.00464

**Published:** 2014-12-04

**Authors:** Brian T. DeCant, Daniel R. Principe, Carmen Guerra, Marina Pasca di Magliano, Paul J. Grippo

**Affiliations:** ^1^Department of Medicine, University of Illinois at ChicagoChicago, IL, USA; ^2^Molecular Oncology Program, Centro Nacional de Investigaciones OncológicasMadrid, Spain; ^3^Department of Surgery, University of MichiganAnn Arbor, MI, USA

**Keywords:** mouse model, pancreatic cancer, inducible, conditional, FLP/FRT, Kras

## Abstract

The study of pancreatic cancer has prompted the development of numerous mouse models that aim to recapitulate the phenotypic and mechanistic features of this deadly malignancy. This review accomplishes two tasks. First, it provides an overview of the models that have been used as representations of both the neoplastic and carcinoma phenotypes. Second, it presents new modeling schemes that ultimately will serve to more faithfully capture the temporal and spatial progression of the human disease, providing platforms for improved understanding of the role of non-epithelial compartments in disease etiology as well as evaluating therapeutic approaches.

## Introduction

There has been a noticeable increase (near doubling) in the 5-year survival of pancreatic cancer patients, though the number remains quite low at about 6–7% (SEER Stat Fact Sheets: Pancreas Cancer—NCI). Much of this stems from a few more potent clinical therapies [folfirinox (Papadatos-Pastos et al., 2014), nab-paclitaxol (Borazanci and Von Hoff, 2014), and various combinations with gemcitabine (Tian et al., 2013)] that improve on previous survival rates. Thus, begins a new drive to employ relevant preclinical models with which to test novel drugs that can further improve patient survival. Indeed, there are already a few mouse models that can be used, with KPC mice (as described below) being one model that currently boasts a strong recapitulation of the paradigm observed in human pancreatic adenocarcinoma. Yet, further advances on mouse models will not only generate additional preclinical models but, perhaps more importantly, demonstrate the utility of newer diagnostic and/or therapeutic targets. The main objective of this review is to highlight past and present mouse models of pancreatic cancer [see (Guerra and Barbacid, 2013) for a more thorough review of current models] in order to propose continued engineering of more relevant mouse systems. These future models could then be employed to better understand the role of non-parenchymal compartments during the development of disease as well as build inducible systems that allow multiple allelic changes at various intervals.

## Transgenic models

Initially, development of cancer in mouse pancreas was demonstrated by targeting Myc and TGFα to mouse pancreatic acinar cells (EL-Myc and EL-TGFα), which demonstrated acinar-to-ductal metaplasia leading to exocrine carcinoma with focally distinct ductal-like lesions (Sandgren et al., 1990, 1991, 1993; Grippo and Sandgren, 2012). Previous targeting of oncogene expression via the elastase (EL) promoter proved effective at inducing exocrine pancreatic neoplasms in transgenic mice, including EL-SV40 TAg and EL-Hras (Ornitz et al., 1985, 1987; Quaife et al., 1987). These two models developed acinar hyperplasia (Ornitz et al., 1987) and carcinoma (Quaife et al., 1987) while EL-TGFα mice produced severe fibrosis, tubular complexes, and aberrant cell morphology (Sandgren et al., 1993). Older EL-TGFα mice eventually develop carcinoma, and tumor development was enhanced in a p53 null background and concomitant with partial or whole loss of INK4a or SMAD4 (Wagner et al., 2001). The metaplasia in EL-TGFα/p53^+/−^ mice was characterized along with its genomic signature (Schreiner et al., 2003) and increased expression of Pdx1, a gene necessary for pancreas development and often expressed in pancreatic cancer, was observed in mice with overexpression of TGFα (Song et al., 1999). Additionally, the EL-KRAS model, which directs human mutant KRAS transgene expression to pancreatic acinar cells via a rat elastase driver, demonstrates a common pancreatic cancer histotype by inducing neoplastic, ductal lesions (Grippo et al., 2003), often referred to as cystic papillary neoplasms (CPNs) similar to human cystic neoplasms including IPMN and MCN (Hruban et al., 2006).

## Conditional models

Conditional systems have become an asset to the mouse-modeling field as they provide tissue specific targeting of genes. One prominent targeting strategy included *Pdx1* and *Ptf1a* or *p48*-driven expression of Cre recombinase in mice with flanking Lox elements (floxed) that, upon Cre-mediated recombination, generated a mutant Kras in the endogenous mouse allele. These mice developed ductal lesions and mPanINs that occasionally progressed to invasive cancer (Hingorani et al., 2003). This model laid the foundation for the generation of the LSL-*Kras*^G12D/+^;LSL-*Trp*53^*R172H*/+^;*Pdx1*−*Cre* (KPC) model which demonstrates a highly metastatic carcinoma that resembles human disease (Hingorani et al., 2005). Models such as this one have allowed for the characterization of biomarkers in pancreatic cancer from disease initiation to metastasis (Mirus et al., 2014). It is important to note that these floxed alleles can be targeted to other cell types in the pancreas as demonstrated by expression of the LSL−*Kras*^*G12D*/+^ allele in Nestin positive cells leading to mPanINs (Carriere et al., 2007) and caerulein-induced PDAC (Carriere et al., 2011b).

Following the use of these models, other conditional targets were generated utilizing similar technology. Since Transforming Growth Factor β (TGFβ) signaling is commonly disrupted in cancer (Principe et al., 2014) and highly so in pancreatic cancer (Jones et al., 2008), LSL-*Kras*^*G12D*/+^;*Tgfbr2^flox/flox^*;*Ptf1a*^*Cre*/+^ mice were generated to simultaneously express mutant *Kras*^*G12D*^ and loss of the type 2 TGFβ receptor (*Tgfbr2*) in pancreatic epithelium. This model demonstrated an aggressive form of pancreatic ductal adenocarcinoma (PDAC) and explored the role of TGFβ signaling in the development of the disease (Ijichi et al., 2006). As loss of downstream TGFβ target SMAD4 is common in pancreatic cancer (Hahn et al., 1996), LSL-*Kras*^*G12D*/+^;*Dpc4*^*flox*/+^;*Pdx*1−*Cre* and LSL-*Kras*^*G12D*/+^;*Dpc*4^*flox*/+^;Ptf1a^*Cre*/+^ were generated to conditionally express *Kras^G12D^* in concert with *Smad4/Dpc4* haploinsufficiency in the pancreas, thereby inducing MCNs and subsequent PDAC (Izeradjene et al., 2007). Additionally, IPMN-like lesions accompanied by PDAC and metastatic disease were shown with the LSL-*Kras^G12D/+^*;*Smad4^flox/flox^;Pdx1-Cre* model (Bardeesy et al., 2006; Kojima et al., 2007).

Considering the implications for loss/inactivation of *p16^Ink4a^* and *p19^Arf^* in cellular transformation, a variety of models have pursued this target in concert with pancreas-specific mutations. An *MT-TGFα;Ink4a/Arf^−/−^* model was generated, ultimately demonstrating a serous cystadenoma (SCA) phenotype that resembled human disease (Bardeesy et al., 2002). Following the creation of this model, pancreas-specific *Kras* targeting was coupled with a floxed *Ink4a/Arf* locus. These LSL*-Kras^G12D^;Ink4a/Arf^flox/flox^;Pdx1-Cre* mice presented with invasive, metastatic disease consistent with human disease (Aguirre et al., 2003). In addition, the LSL*-Kras^G12D/+^;p16^flox/flox^;Pdx1-Cre* model directed the knockout of the *p16^Ink4a^* tumor suppressor gene in pancreatic epithelium. These mice developed mPanINs, PDAC, and metastases (Qiu et al., 2011). Characterization of this tumor suppressive axis also prompted the generation of LSL*-Kras^G12D/+^;Rb^flox/flox^;Pdx1-Cre* mice to assess the role of *Rb* inactivation and PDAC progression. These mice exhibited accelerated mPanIN progression and rapid PDAC development (Carriere et al., 2011a).

The activation of mutant *Kras* and heparin-binding epidermal growth factor-like growth factor (*HB-EGF*) by the Means group also demonstrated conditional targeting of two oncogenic events. These mice featured rapid progression into the early stages of pancreatic cancer (Ray et al., 2014).

The tumor stroma's control of tumor growth was explored by utilizing two conditional models of pancreatic cancer. *Shh^flox/flox^*;*Pdx1-Cre*;LSL-*Kras*^*G12D*/+^;*p*53^*flox*/+^;*Rosa*26^*LSL*−*YFP*^ (ShhPKCY) mice were generated to delete Sonic Hedgehog (SHH) in the context on PDAC. Due to lack of SHH, these mice presented with less tumor stroma yet more aggressive, proliferative tumors. This phenotype was also shown utilizing a Smoothened inhibitor in KPC mice. Additionally, VEGFR inhibition promoted SHH-deficient tumor survival, demonstrating that SHH-formed stroma limits tumor growth by restricting tumor angiogenesis. (Rhim et al., 2014).

Additional study of the tumor stroma's contribution to cancer growth was explored via the generation of a mouse model that crosses LSL*-Kras^G12D/+^*;*Tgfbr2^flox/flox^*;*Ptf1a^Cre/+^* mice to αSMA-tk transgenic mice. Depletion of αSMA^+^ myofibroblasts in the context of mPanINs or PDAC resulted in reduced survival characterized by hypoxia, EMT, and cancer stem cells. In addition, this model was characterized by the increase in regulatory T cells infiltrating myofibroblast-depleted tumors. Similar results were shown when the KPC model was used in cross with the αSMA-tk transgenic (Ozdemir et al., 2014).

Both of these studies hold implications for the future of stromal-directed therapies for the treatment of PDAC. Although mouse models have been successful for such therapies (Olive et al., 2009), the recapitulation of these results in clinical trials has largely failed. Rhim and Ozdemir demonstrated that tumor stroma provided a protective effect for the host. Therefore, targeting the stroma may create a more aggressive form of PDAC. As noted by Gore and Korc, the stroma's capacity for both benefit and damage must be further explored in mouse models before potential therapies are reapplied in human trials (Neesse et al., 2011; Gore and Korc, 2014). However, ablation of a subpopulation of stromal cells (FAP+ cells) permitted immune control of tumor growth and uncovered the efficacy of immunotherapeutic antibodies (anti-CTLA-4 or anti-PD-L1), which resulted in acute tumor regression (Kraman et al., 2010; Feig et al., 2013). More recently it has been shown that VDR acts as a master transcriptional regulator of PSCs to reprise the quiescent state, resulting in induced stromal remodeling, increased intratumoral gemcitabine, reduced tumor volume, and a 57% increase in survival compared to chemotherapy alone (Sherman et al., 2014). The distinct outcome of these studies underscores the need to better understand the role of desmoplastic stroma in pancreatic cancer.

## Inducible/conditional mouse modeling systems of pancreatic cancer

While the described conditional modeling systems have provided invaluable insight into disease incidence and progression, they do not fully capture the temporal component of human mutations observed in the clinic. For instance, in systems relying on *Pdx* or *Ptf1* driven Cre, recombination occurs at E8.5 (Ohlsson et al., 1993) or E9.5 (Obata et al., 2001), respectively. While embryonic recombination often shortens the time to a cancer or neoplastic phenotype, the effects of these mutations on pancreatic development are not fully understood, and do not faithfully mimic the spontaneous mutations that occur in the fully formed gland of an adult human patient.

In recent years, conditional and inducible systems have prompted the unique ability to control when and where genes are expressed. In particular, the development of CreERT technology (Feil et al., 1996, 1997) has prompted an array of tissue specific, temporally-controlled targeting models. Both the *ElastaseCreERT2* (Desai et al., 2007) and the *Ptf1a^Cre-ERTM^* (Kopinke et al., 2012) systems have advanced the field of pancreatic cancer modeling by providing a means for inducibly targeting pancreatic epithelium. Both of these systems feature a Cre recombinase cassette fused to a Tamoxifen-responsive mutant estrogen-receptor element that is driven by an acinar cell specific promoter region. The Cre recombinase in each of these systems is then able to activate gene expression in a loxP-mediated system. The utility of the CreERT system was further demonstrated in the *Kras^G12D^*;*Rosa26^NIC^*;*Pdx1-CreERT* model, which temporally controlled the expression of *Notch* and *Kras* and showed synergistic effects between the two proteins with respect to mPanIN progression (De La et al., 2008).

### iKras^*^ models

The Pasca di Magliano group has also generated several models that represent the full utilization of both spatial and temporal control of gene expression. The iKras^*^ model functions through the transgenesis of three different types of mice. In these mice, the *Ptf1a* allele drives Cre expression (Kawaguchi et al., 2002), which, in turn, excises a stop cassette bound by two loxP sites. This stop cassette functions to inhibit the reverse tetracycline transactivator (rtTa) for an IRES-EGFP cassette at the R26 locus (Belteki et al., 2005). Since *Ptf1a^Cre/+^* is mostly pancreas specific, the excision of the stop cassette allows for the expression of both rtTa and EGFP in the pancreatic epithelium beginning during embryogenesis (Collins et al., 2012a).

Administering doxycycline to these animals leads to activation of rtTa and subsequent Kras^*^ expression through a TetO-*Kras^G12D^* transgene using rat mutant *Kras* (Fisher et al., 2001). This inducible system provides a strong platform to explore several relevant issues. First, the mutation of *Kras* can be expressed in adult tissues, which is far more relevant to PanIN progression to cancer observed in humans. In addition, it allows for the abrogation of oncogenic *Kras* expression at various stages of cancer development and thus the study of the dependence of developing lesions and cancer on mutant *Kras*. Also, this system can be employed to investigate carcinogenesis in the context of tumor suppressor inactivation or additional oncogene activation. iKras^*^-p53^+/−^ mice were also generated to illustrate the development of PDAC when mutant *Kras* is paired with the concurrent inactivation of this tumor suppressor gene (Collins et al., 2012a). This model provides a framework examining various features of oncogenic *Kras* in PDAC development. Inhibition of mutant *Kras* expression through doxycycline removal and subsequent reversion to a more normal phenotype supports continued efforts to target mutant *Kras* as a therapeutic option and eventual translation to the clinic.

Furthermore, the Pasca di Magliano group generated a model that inducibly and conditionally activated *Kras* and a mutant p53 allele (Collins et al., 2012b). These mice featured the same iKras^*^ system described above with an additional mutant p53 allele preceded by a loxP-bound STOP cassette. Therefore, the same *Ptf1a*-driven Cre-recombinase that activates the rtTa for iKras^*^ expression will also activate the mutant p53^R172H^ (p53^*^) allele (Olive et al., 2004) by excising the preceding STOP cassette. However, in these iKras^*^p53^*^ mice, oncogenic *Kras* is not activated until doxycycline administration. This model demonstrated a dual functionality by allowing the simultaneous, pancreas-specific targeting of two alleles (iKras^*^ and p53^*^) and the inducible/reversible expression of oncogenic *Kras* (Collins et al., 2012b). Although the conditional LSL*-Kras^G12D/+^;*LSL*-Trp53^R172H/+^;Pdx1-Cre* (KPC) model (Hingorani et al., 2005) of PDAC demonstrated a close mimicking of the human disease, it lacked inducible control of *Kras*. This type of control over mutant *Kras* expression allowed for the study of its role in primary and metastatic tumor maintenance when expressed concurrently with mutant p53 (Collins et al., 2012b) and the demonstration of mutant *Kras*-dependence on more aggressive and metastatic pancreatic cancer.

### LSL-*Kras^+/G12Vgeo^*;*EL-tTA/tetO-Cre* models

Additionally, the Barbacid group generated a model that accomplishes both temporal and spatial targeting of oncogenic Kras using a different mutant variant (G12V vs. G12D). By crossing a LSL*-Kras^+/G12Vgeo^* knockin strain (Guerra et al., 2003) to EL-tTA/tetO-Cre mice, their group was able to obtain an inducible system of endogenous *K*ras^G12V^ mediated by doxycycline control of Cre recombinase activity (Guerra et al., 2007). Essentially, removing doxycycline in this tet-off system permits an elastase-driven Cre specific to acinar and centroacinar cells of the pancreas. The Cre changes LSL-*Kras^G12Vgeo^* into the active, oncogenic *Kras^G12Vgeo^* by excising the loxP sites that contain a stop cassette. The utility of this system is further advanced by the detection of cells that ultimately end up with *Kras^G12V^* expression. A knockin of IRES-*geo* into the 3′ untranslated sequences of the *Kras* allele allows for LacZ expression when the LSL cassette is removed (Guerra et al., 2003). LacZ encodes β-galactosidase, which is then detectable via histochemical staining. Initially, this system was used to induce expression of oncogenic *Kras* at E16.5, leading to the production of mPanIN lesions that could advance in severity following caerulein administration (Guerra et al., 2007). Surprisingly, doxycycline removal in adult stages resulted in widespread expression of *Kras^G12V^* in adult acinar cells with no phenotypic consequences. Interestingly, adult mice that express *Kras^G12V^* in the acinar cell compartment develop mPanINs and PDAC in the context of pancreatitis.

To explore the resistance of postnatal acinar cells to transformation via the expression of *Kras*, the Barbacid group also characterized the role of several tumor suppressors. These acinar cells were resistant to transformation even in the absence of tumor suppressors. *Kras^+/G12V^*;p16*Ink4a*/p19*Arf*^*flox/flox*^;*EL*-tTA/*tetO*-Cre and *Kras^+/G12V^*;*Trp*53^*flox/flox*^;*EL*-tTA/*tetO*-Cre mice were generated and given doxycycline from birth until P60 (Guerra et al., 2011). Acting under the same tet-off system as described above, these mice, when taken off doxycycline, were subject to expression of Cre recombinase in acinar and centroacinar cells of the pancreas. However, instead of just activating *Kras*, the Cre simultaneously excised the floxed p16*Ink4a*/p19*Arf* or *Trp*53 alleles. These models, when combined with caerulein-induced pancreatitis, presented an invasive, metastatic PDAC phenotype (Guerra et al., 2011).

### TVA-RCAS models

Another mouse model system that features viral delivery for eventual induction of gene expression or loss of cell targets demonstrates the versatility of this field and another avenue for creating complex inducible/conditional schemes. Varmus and colleagues generated a model that introduced a replication-competent avian leukosis sarcoma virus long-terminal repeat with splice acceptor (ALSV-A-based RCAS) vector to mice that expressed the ALSV-A receptor, TVA, (Orsulic, 2002) under the control of the elastase promoter (Lewis et al., 2003). This elastase-*tva* model allowed somatic acinar cells of the pancreas to incorporate RCAS-delivered genes, such as polyoma virus middle T antigen (PyMT) (Gottlieb and Villarreal, 2001) or c-Myc, into the host cell genome. These elastase-*tva* mice were crossed to *Ink4a/Arf* null mice to create models characterizing the phenotype resulting from these initiating oncogenic events (Lewis et al., 2003). They found that PyMT and c-Myc induced different types of pancreatic tumors, illustrating the impact of the initiating lesion on resulting tumor pathology.

The development of this TVA-RCAS model was further expanded with the coupling of the elastase-*tva* mice with *Trp53* flox;*Ptf1a-Cre* (Jonkers et al., 2001) (Kawaguchi et al., 2002) mice (Morton et al., 2008). In this model, delivery of the PyMT oncogene is accompanied by the pancreas-specific deletion of the tumor suppressor, *Trp53*. Results of this model showed metastatic disease to the liver. In addition, the elastase-*tva*;*Trp53*^*flox/flox*^;*Ptf1a^Cre/+^* mice were crossed to *Ink4a/Arf*^*flox*/+^ (Krimpenfort et al., 2001) mice to assess tumor development in the context of a simultaneous p53 deficiency and *Ink4a/Arf* single allele deletion. Results of this model elucidated a much more aggressive tumor model after PyMT activation via virus administration (Morton et al., 2008). This model succeeds as an example of both conditional and temporal control of gene expression by combining both pancreas–specific deletion of *Trp53* via Cre-recombinase activity and acinar cell-directed, inducible PyMT expression via elastase-*tva* targeting.

Lewis and his group expounded upon these findings by crossing the *elastase-tva* model with LSL*-Kras^G12D^;Ptf1a^Cre/+^* mice (Hingorani et al., 2003) to assess the impact of activated Wnt signaling in the context of KRAS-induced pancreatic tumorigenesis (Sano et al., 2014). These mice were injected with chick fibroblasts that produced ALSV-A-based RCAS vectors encoding Wnt1 or a GFP control, ultimately resulting in host genome uptake of these genes in pancreatic acinar cells and their progenitors. Thus, this model allowed for the targeting of Wnt1 to the pancreatic epithelium and subsequent characterization of its signaling activity when introduced in concert with *Kras* activation. They found that in this context, activated Wnt signaling induced the formation of mucinous cystic neoplasms (MCN). Interestingly, these mice displayed higher Wnt signaling in the stroma of the MCNs, rather than in the cyst epithelium, which is consistent with MCN patient data (Sano et al., 2014). These results suggest that Wnt ligands may act in a paracrine fashion to stimulate MCN development.

## Exploring inducible/conditional systems coupled with epigenetic events

The significance of factors external to genomic changes in these models must not be overlooked. Multiple mutant *Kras*-expressing models have demonstrated the contribution of inflammation and dietary aspects to pancreatic cancer pathogenesis, improving our understanding of pancreatic cancer and pancreatitis as well as the interplay between the two. It was shown that high levels of Ras activity in cLGL-*Kras^G12V^*;EL*-CreERT* generated high levels of fibrosis and inflammation that mimicked chronic pancreatitis. Since elevated Ras activity is also found in PDAC, this finding provided a mechanistic link between pancreatic cancer and chronic pancreatitis (Ji et al., 2009; Logsdon and Ji, 2009).

Other mechanisms have been explored with respect to inflammatory insult and subsequent neoplastic and cancerous phenotypes. Utilizing a breadth of models, Jack's group established that chronic pancreatitis may provide enough insult for insulin-expressing endocrine cells to become susceptible to KRAS-induced transformation (Gidekel Friedlander et al., 2009). Logsdon and colleagues also demonstrated that with caerulein induction of acute pancreatitis in the presence of inducible mutant *Kras* (LSL-*Kras^G12V^*;EL-*CreERT*) there was NF-κB mediated amplification of Ras activity. These mice presented with chronic inflammation and mPanIN lesions that subsided with the inhibition of Cox-2 or deletion of *IKK2* (Daniluk et al., 2012). This effect was also demonstrated in KC mice with loss of Cox-2 despite the additional loss of pTEN, highlighting the potential role of AKT activation in chemoresistance (Hill et al., 2012). Likewise, the LSL-*Kras^G12V^*;EL-*CreERT* model was used in a cross with Cox-2 conditional knockout mice to study the effects of high fat diets on PDAC. LSL-*Kras^G12V^*;EL-*CreERT* mice fed high fat diet presented with increased fibrosis, mPanINs, and PDAC compared to no increased mPanIN lesions or PDAC in COX^flox/flox^;LSL-*Kras^G12V^*;EL-*CreERT* mice fed the same diet (Philip et al., 2013). Similarly, KC mice were shown to generate mPanIN lesions at an earlier onset following a high fat, high calorie diet with a subsequent increase in infiltration of macrophages and T cells in an expanded stromal bed (Dawson et al., 2013).

Progression of mPanINs and PDAC has also been explored in the context of inhibitors to the Ras signaling pathway. Gefitinib, an EGFR inhibitor, was given to LSL-*Kras^G12D/+^*;*Ptf1a^Cre/+^* mice, demonstrating a prevention of mPanIN and PDAC development (Mohammed et al., 2010). Similarly, it was shown that inhibition of EGFR does not allow for RAS levels sufficient for the transformation seen in PDAC (Ardito et al., 2012; Navas et al., 2012).

## Future applications of inducible/conditional modeling systems

The mouse-modeling field has capitalized on conditional and/or inducible Cre-lox technology to target gene expression in numerous cell types. However, the overwhelming majority of pancreatic cancer models rely on Cre-lox to drive oncogenic *Kras* in the pancreatic epithelium, excluding the use of non-epithelial Cre systems and limiting the ability to target other cells types involved in carcinogenesis. Therefore, utilizing non-Cre-lox driven systems to target mutant *Kras* to pancreatic epithelium will allow compatibility with a vast array of preexisting Cre-lox systems that target genetic changes to additional cell types including the stroma and hematopoietic cell compartments.

The use of single transgenic or knockin systems in combination with Cre-lox models that target non-parenchymal cells in the pancreas can circumvent some of the limitations that arise when using Cre-lox to drive an initiating event like mutant *Kras*. The EL-KRAS model may be a prime candidate for combined Cre-lox targeting of other cell types, as these mice develop acinar-to-ductal metaplasia and cystic papillary neoplasms (CPN) that resemble human cystic disease in the pancreas. These lesions did progress to PDAC in a p16 null background or acinar carcinoma when in a p53 null background (personal communication with Dr. Eric Sandgren).

EL-TGFα (Sandgren et al., 1990) and *Mist1^KrasG12D/+^* (Tuveson et al., 2006) models can serve as potential neoplastic drivers used in concert with Cre-lox targeting. EL-TGFα mice have been employed in combination with p53 loss (Greten et al., 2001; Schreiner et al., 2003) to generate a model of advanced pancreatic cancer with hallmark genetic features (loss of p16, inactivation of Cdkn2a) reminiscent in human disease and, in combination with mutant *Kras*, development of CPN that resembles human IPMN (Siveke et al., 2007). EL-TGFα does lead to proliferation of acinar cells and fibroblasts and focally generated metaplastic lesions derived from acini (Sandgren et al., 1990). Yet, there was no reported observation of neoplasia or more advanced lesions in this model. *Mist1^KrasG12D/+^* mice developed a predictable lethal pancreatic cancer phenotype characterized by acinar metaplasia and dysplasia in its early stages (Tuveson et al., 2006). Despite being a strong model of the pancreatic neoplasia to cancer paradigm as an ectopic model of mutant *Kras* expression, *Mist1^KrasG12D/+^* mice did, rather unexpectedly, develop hepatocellular carcinoma (Tuveson et al., 2006). This feature of the model may be of potential concern when attempting to evaluate the phenotypes of genetically engineered mice that employ this particular initiating event. However, an inducible targeting of LSL-*Kras^G12D/+^* with *Mist1^CreERT2/+^* produced mPanIN lesions, indicating the relevance of the *Mist1*-expressing compartment in the origins of PDAC (Habbe et al., 2008). Although EL-KRAS mice do, on occasion, develop PanIN-like lesions, these are not the predominant histotype in the pancreas, as PanIN lesions are more frequently observed in human disease. Nonetheless, these transgenic approaches are compatible with non-mutant Kras driving Cre-lox systems and may prove useful in understanding disease etiology in combination with genetic manipulations in other cell compartments. These models do have utility with future approaches, though they lack recapitulation of the predominant clinical histotype (PanIN to PDAC).

Therefore, a FLP/FRT KRAS model poses the most promise for inducing *Kras* mutations that result in a PanIN-like phenotype while allowing the use of Cre-lox to target different genetic events in other cell types. In a manner similar to the Cre-lox system, FLP/FRT utilizes a recombinase called flippase to target FLP recombinase targets that flank an endogenous gene (Dymecki, 1996). Unlike Cre, which is derived from P1 bacteriophage, the FLP recombinase is derived from *Saccharomyces cerevisiae* (Sadowski, 1995). Ideally, a desirable model would involve the generation of a pancreas-specific FLP directed toward a FRT target sequence that flanks a stop codon upstream of oncogenic *Kras*. At this point, a pancreas-specific FLP may be possible with the intraductal injection of an adenovirus FLP or the generation of an EL-tTA;TetO-FLP;FSF-*Kras^G12D/+^* mouse. Ideally, this mechanism would drive mutant *Kras* in a near identical fashion as *EL-Cre*;LSL-*Kras^G12D/+^* while still allowing for the targeting of non-epithelial cell types with Cre-lox.

While this type of model would increase our understanding of the contributions of stromal, hematopoietic, and other cell types to pancreatic carcinogenesis, the ultimate goal of such a system would be the design of a layered model that is simultaneously and/or sequentially inducible. Mimicking a temporal progression of gene mutations in specific cellular compartments requires the use of multiple systems employing different modes of induction. As described, the CreERT system has been well established for many gene targets but alone can only deliver multiple mutations simultaneously (Frese and Tuveson, 2007). Young and colleagues demonstrated the potential of the FLP/FRT system when coupled with Cre-lox in lung tissue. They generated mice with an Flp inducible allele of *Kras^G12D^* and Cre driven mutation of the tumor suppressor, *p53* (Young et al., 2011). The FLP-FRT system, FSF-*Kras^G12D^*, was induced through an adenovirus or lentivirus expressing Flpo, a version of Flp optimized for mammalian use. Utilization of this mammalian version of Flp, as opposed to Flpe, was utilized due to its higher recombination efficiency (Farley et al., 2000). Injection of the adenovirus/lentivirus activates mutant *Kras* and results in numerous lung tumors, ultimately confirming that FSF-*Kras^G12D^* results in a phenotype similar to LSL-*Kras^G12D/+^*allele. This virus-driven FLP-FRT was coupled with a tamoxifen-driven p53 mutation via Cre recombinase activity (Young et al., 2011).

The TVA-RCAS targeting of epithelial tissue and subsequent stromal phenotype indicates further opportunity for the utilization of this system to target other cell types simultaneously. For example, the conditional nature of this model would allow for the targeting of genes to the stroma via a TVA-RCAS system utilizing a driver such as αSMA or Vimentin. Taking this further, the possibility arises for generation of a trigenic model. Utilizing Cre-lox, FLP/FRT, and TVA-RCAS targeting methods in the same mouse would provide a novel way to target several different cell types in both a conditional and inducible manner.

## Advancing the utility of inducible/conditional modeling

While the aforementioned models are undoubtedly technological achievements, their ability to faithfully recapitulate human disease is still limited. Clinically, at least two gene mutations occur to produce PDAC. *Kras* is believed to be the first mutation in a series of transformation events that lead to PDAC in adults. Subsequent major mutations include those to p53, SMAD4, or p16^INK4a^, among several others (Hezel et al., 2006). With current mouse models, recombination events affecting *Kras* and these other genes occur either during embryonic development or concomitantly sometime after pancreas formation, in the case of inducible systems. However, they fail to capture the step-wise mutation process that occurs in the adult pancreata of human patients.

**Figure 1:**
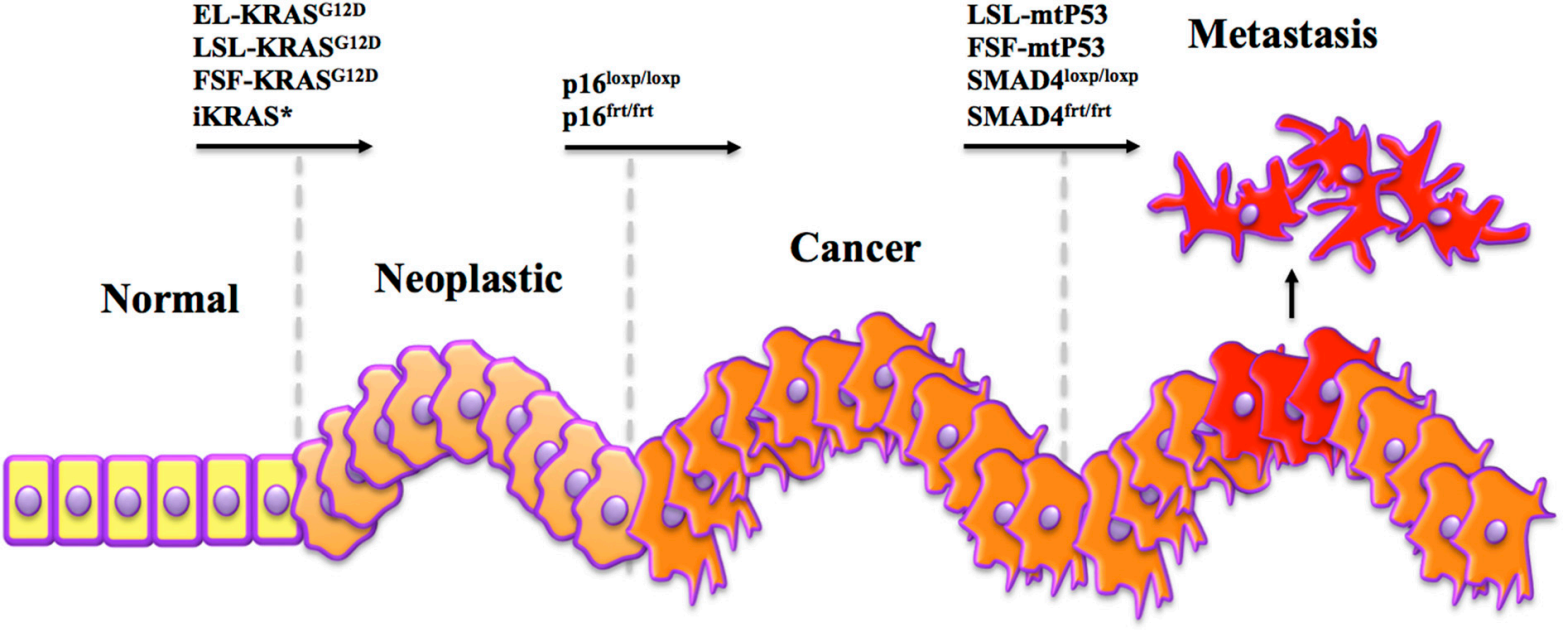
**Mimicking human tumorigenesis through temporal modeling of pancreatic cancer**. A key difference between human pancreatic cancer and commonly used mouse models is in the timing of mutations. In human patients, *Kras* mutations are often considered an initiating event, occurring in adult cells, soon followed by mutations to *p16*, and later *p53* and/or *SMAD4*. Yet in most models, *Kras* and altered tumor suppressor genes are induced simultaneously in the developing embryo. Despite a human-like histotype, these models have yet to be accurate predictors of outcomes observed in clinical trials. Therefore, we propose that using combinations of several systems to drive sequential *Kras, p16*, and *SMAD4/p53* mutations may lead to more human-like disease that responds to therapy more like that observed in the clinic.

**Figure 2:**
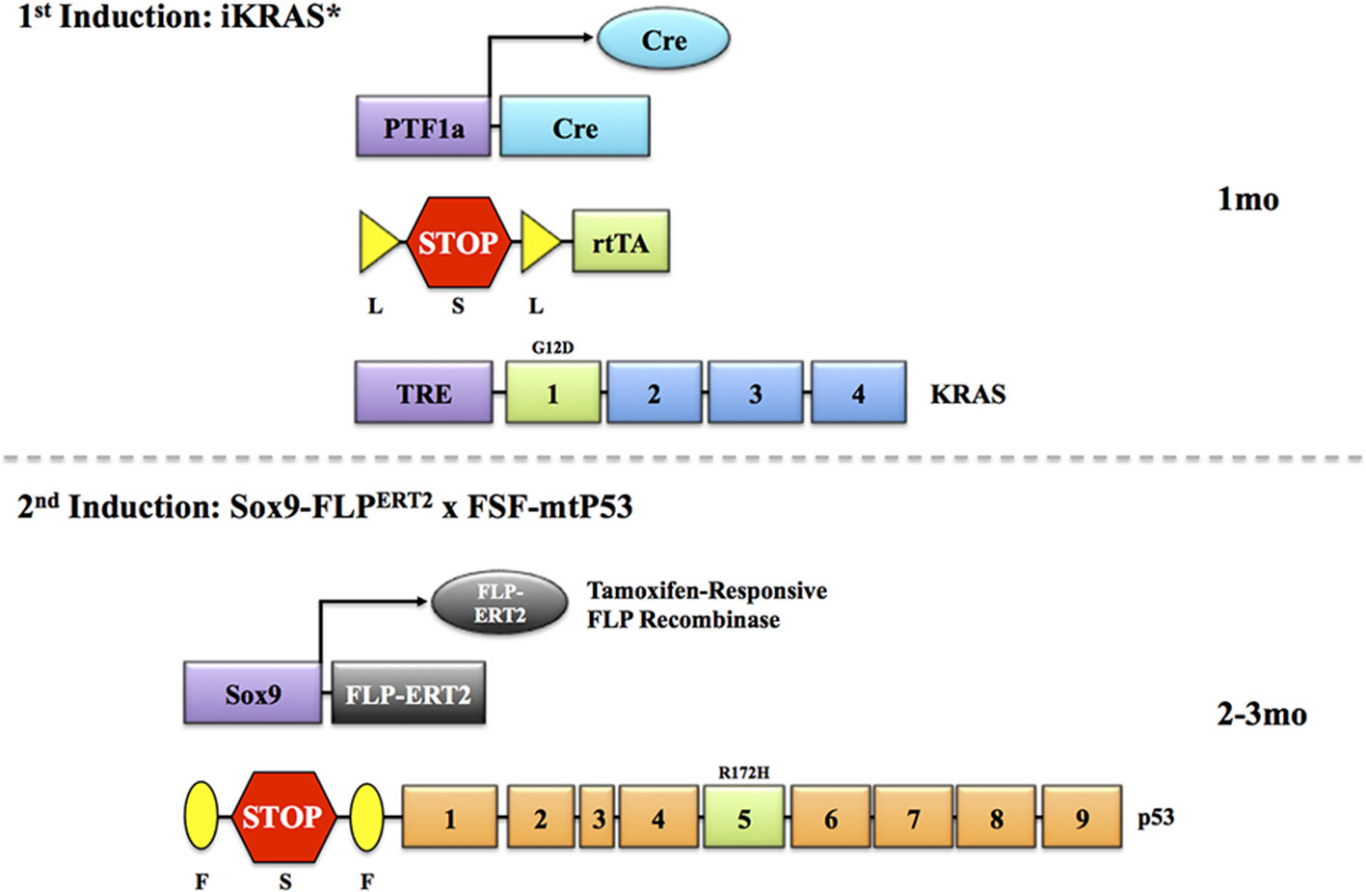
**Temporal modeling via two inducible systems**. In order to address the issue of successive induction of mutations as they occur in human, several modeling systems can be employed. In this example, as designed by the Pasca di Magliano group, expression of Cre-recombinase is driven by the *Ptf1a* promoter. This is combined with a LSL cassette followed by an rtTA sequence. In the presence of Cre, the stop codon is excised, and rtTA is transcribed. This allows for interaction with a third transgene, a TRE-*Kras*. When doxycycline is administered, oncogenic *Kras* expression is induced. By activating this system at 1 month, it would allow a simulated *Kras* mutation in near-adult tissues. Once lesions manifest, this can be followed by the induction of a second transgene, a mutant *p53* driven by a *Sox9*-FLP^ERT2^ recombinase. This will excise a stop codon in front of a mutant *p53* sequence in the presence of tamoxifen, and drive mutant *p53* expression. The p16 allele could also be engineered in the same manner. Timing of these events will likely have to be determined empirically, as mutant *Kras* expression in adult pancreas may not lead to the development of neoplastic lesions without an external stimulus (like caerulein). Indeed, a third allelic alteration may be necessary to drive a more aggressive metastatic phenotype (see Figure 3).

**Figure 3:**
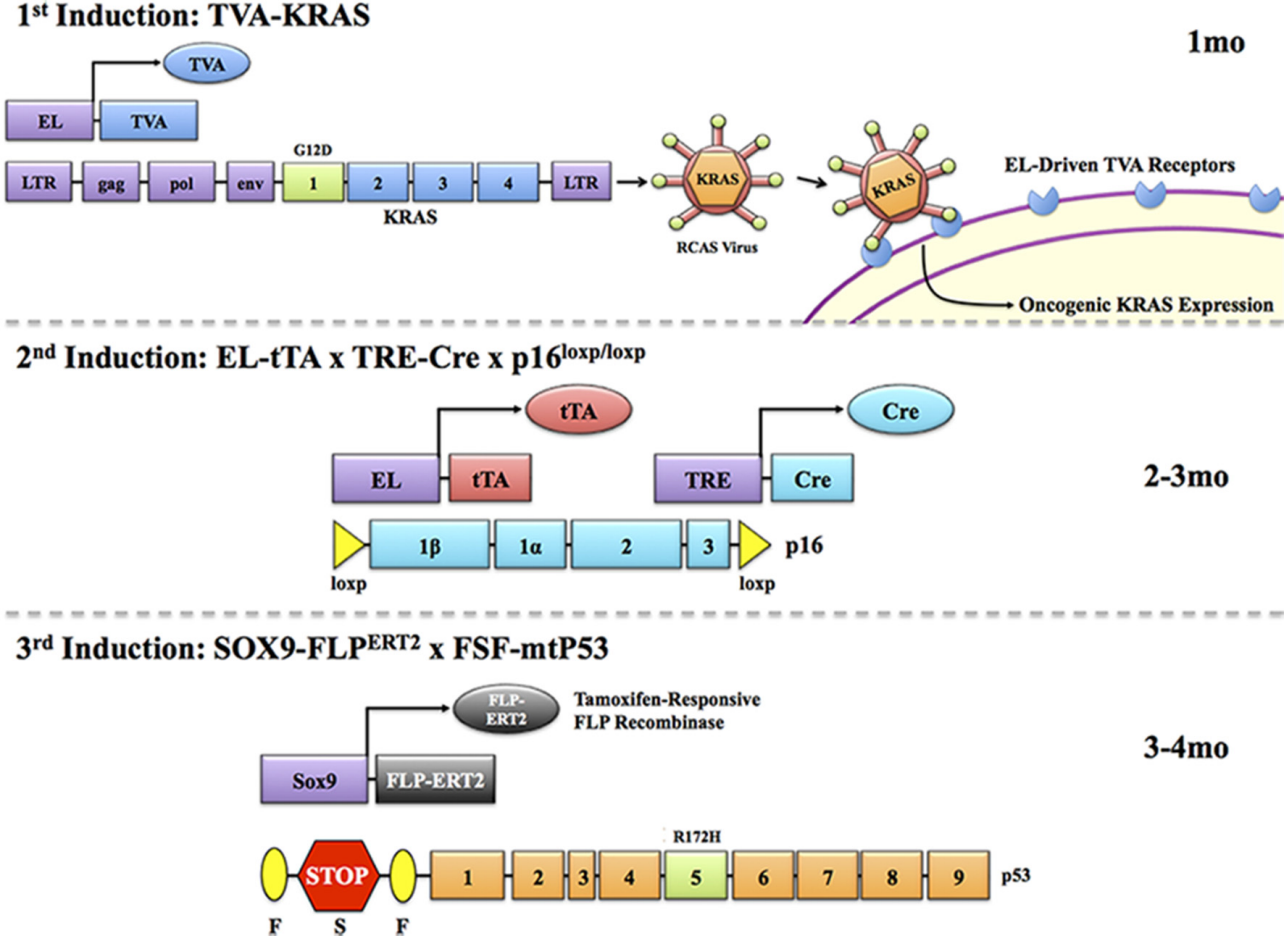
**Temporal modeling via three inducible systems**. As human malignancies often involve several mutations, a compound inducible system may be employed to target three successive transgenes to the same cell type. For example, mt*Kras* may be first induced through a TVA/RCAS virus system. In this system, expression of a TVA receptor is targeted to the pancreas via the elastase promoter. Upon reaching adulthood, animals can be administered a RCAS virus coding for the mt*Kras* gene. This will interact only with cells expressing the TVA receptor, allowing for targeted and inducible expression of KRAS in the pancreas. A second mutation, such as loss of p16, can then be induced in the same cells via an elastase driven tTA that, in the presence of doxycycline, will induce expression of Cre through TRE-Cre. Combining this with a p16^flox/flox^ gene will allow for doxycycline-induced loss of the p16 gene, and the second genetic hit. Finally, a tamoxifen-responsive *Sox9*-FLP^ERT2^ can target cells expressing ductal markers (including those having undergone acinar-ductal metaplasia), allowing for inducible expression of mtP53 via an FSF cassette, providing the third genetic hit as it often occurs in humans. It is important at each induction point that promoter/gene regulatory elements employed to run the next step be evaluated in the previous model. Hence, acinar-specific markers (eg., Amylase) should be assessed in pancreas following mutant *Kras* expression (TVA/RCAS delivery) and Sox9 antibodies should be used to demonstrate Sox9 expression in mtKras expressing pancreas with loss of p16. This would need to be done at the empirically derived time points (times provided in this figure are merely considerations) when the next induction is scheduled to begin.

Layering multiple inducible systems to target the same cell type and cause multiple mutations in a step-wise manner would assist in capturing a more faithful representation of human disease progression (Figure 1). For example, targeting *Kras* with an EL-tTA or EL-TVA system would provide a mechanism for issuing the first hit of genetic instability in both a temporal and tissue-specific manner. However, it should be noted that elastase targeting in these systems may be dramatically inefficient after pancreas cells advance to a ductal and/or abnormal phenotype. Ablation of a second gene such as *p53, SMAD4*, or p16^*INK4a*^ could then be controlled by a Cre-ERT2 system directed toward the same cells expressing mutant KRAS (Figure 2). Finally, a third system, the FLP/FRT, could be utilized to mutate a third gene in an effort to drive metastatic phenotypes. This trigenic model, which is just one example of many possible inducible/conditional mutation schemes, would better serve to mimic the progressive nature of PDAC (Figure 3). However, generation of such models inherently results in very complex breeding patterns. Additionally, once these trigenic mice are established the induction of different mutations requires a labor-intensive injection scheme and administration of doxycycline over extended periods of time.

**Figure 4:**
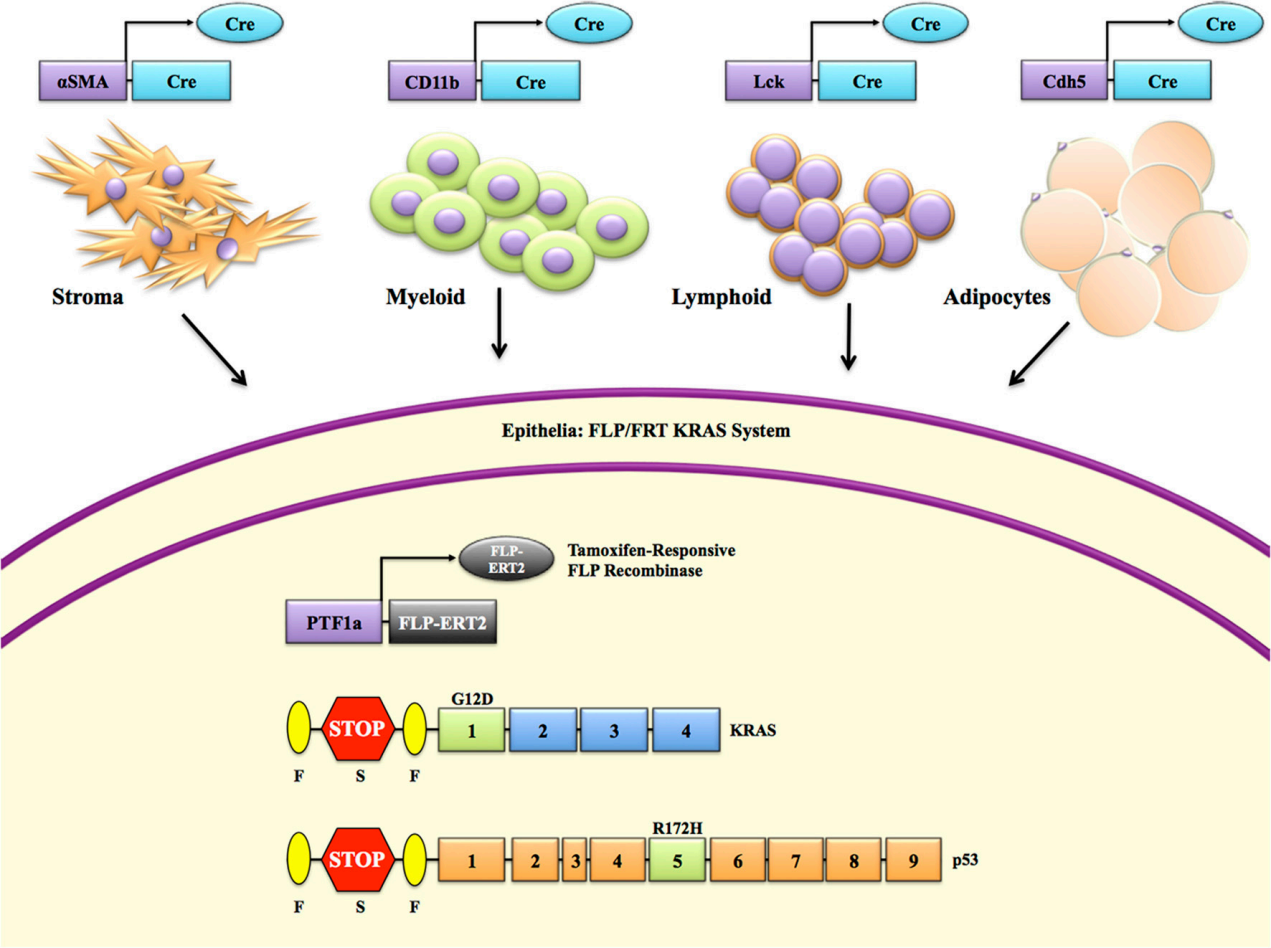
**Spatial modeling of pancreatic cancer to explore cross compartmental interactions**. Cre-loxP is the most widely used conditional targeting system. This is also true in models of pancreatic cancer, where it is primarily used to drive mtKRAS via a loxP-stop-loxP (LSL) cassette. However, reliance on Cre-loxP to induce a *Kras* mutation limits our ability to target other pertinent cell types in the tumor microenvironment. Should mt*Kras* be induced by another system, for example a *Ptf1a*-FLP-driven Frt-stop-Frt (FSF) cassette, which would allow compatibility with one of the several hundred possible Cre-loxP combinations. For instance, an *αSMA-Cre* to explore the contributions of pancreas stellate cells to tumorigenesis, *CD11b-Cre* to target myeloid cells, *Lck-Cre* to target lymphoid cells, or *Cdh5-Cre* to target mature adipocytes (See Table 1).

From a functional standpoint, the utilization of inducible/conditional drivers other than Cre recombinase for the activation of mutant KRAS allows for subsequent Cre-lox targeting of cell types outside the epithelial compartment (Figure 4). Strategically, withholding Cre-lox targeting of *Kras* encourages the use of abundant, pre-existing Cre-lox systems (Table 1) that can target stromal, hematopoietic, and adipose compartments. However, this type of modeling is not necessarily relevant from a clinical standpoint, due lack of evidence that these non-epithelial mutations are common in human PDAC. Nevertheless, this approach allows for more rigorous evaluation of the contributions that different components of the tumor microenvironment (TME) have on carcinogenesis. Insight into the mechanism behind TME involvement in tumor progression and metastatic phenotypes may provide strategies and the rationale for targeting these compartments with certain therapeutic agents. These inducible/conditional systems will be highly relevant in studying the therapeutic value of a genetic target in mature tumors and not at the initiation stages. For instance, a model with expression of oncogenic *Kras^G12V^* and deletion of p53 with an EL-tTA FLP system used in conjunction with ablation of a target gene, such as EGFR, by an ubiquitous Cre-ERT2 system is under development in the Barbacid laboratory.

**Table 1:** **Tissue Specific Cre-lox Targeting Systems**.

**Compartment**	**Cell/Tissue Type**	**Targeting Model**	**Reference**
Epithelium	Pancreatic epithelium, antral stomach, and duodenum in neonates. Pancreatic beta islet cells in adults.	*Pdx1-Cre*	Hingorani et al., 2003
	Pancreatic acinar cells	*ElastaseCreERT2*	Desai et al., 2007
	Pancreatic acinar cells	*p48-Cre Ptf1a^Cre/+^ Ptf1a^Cre-ERTM^*	Hingorani et al., 2003; Kopinke et al., 2012
	Pancreatic acinar cells	*Mist1*^*Cre-ERT2*/+^	Tuveson et al., 2006
Mesenchyme	Myofibroblast	*αSMA-Cre*	Wu et al., 2007
	Myofibroblast	*Vim-Cre*	Troeger et al., 2012
	Smooth muscle	*SMA-CreERT2*	Wendling et al., 2009
	Interstitial stroma of mature tissues—prostate, forestomach, skin	*Fsp1-Cre*	Bhowmick et al., 2004; Teng et al., 2011
	Bone, cartilage	*Dermo1-Cre Twist2-Cre*	Yu et al., 2003; Chen et al., 2008; Liu et al., 2010
	Pancreatic exocrine lineages	*Nestin-Cre*	Delacour et al., 2004
	Dermis, lung, pericardial connective tissue, blood vessel wall, splenic capsule, mesangial cells of glomerulus	*Col1a2-CreERT*	Zheng et al., 2002; Riopel et al., 2013
	Nestin-negative mesenchymal progenitors	*Prx1-Cre*	Greenbaum et al., 2013
Hematopoietic	CD4+ T Cells	*CD4-Cre*	Tanigaki et al., 2004
	Peripheral CD8+ T Cells	*CD8a-Cre*	Maekawa et al., 2008
	Liver and T lymphocytes after IFN or pI-pC induction	*Mx1-Cre*	Alonzi et al., 2001
	Myeloid lineage	*Cd11b-Cre*	Boillee et al., 2006
	Macrophages, granulocytes, possibly other myeloid derived cells	*LysM-Cre*	Clausen et al., 1999
	T lymphocytes and thymocytes	*Lck-Cre*	Tomita et al., 2003; Choi et al., 2013
	Hematopoietic cell lineages to peripheral blood, bone marrow, and spleen [Ectopic expression in PDAC (Fernandez-Zapico et al., 2005)]	*Vav1-Cre*	Daria et al., 2008
	Neutrophils, monocytes/macrophages, some dendritic cells	*Lactotransferrin-Cre*	Kovacic et al., 2014
	Hematopoietic stem cells/progeny	*Pf4-Cre*	Calaminus et al., 2012
	Immature B lymphocytes	*CD19-Cre*	Zhang et al., 2012
	Lymphoid and granulocyte-monocyte progenitors	*Flt3-Cre*	Buza-Vidas et al., 2011
Adipose	Brown and white adipose tissue	*aP2-Cre FABP4-Cre*	Cole et al., 2012
	Brown and white adipose tissue	*aP2-CreERT2*	Dali-Youcef et al., 2007
	Muscle, white adipose tissue, brain	*GLUT4-Cre*	Lin and Accili, 2011
	Brown and white adipocytes, skeletal muscle, dermis	*Myf5-Cre*	Sanchez-Gurmaches and Guertin, 2014
	Brown and white adipose tissue	*Adipoq-Cre*	Berry and Rodeheffer, 2013
	Mature adipocytes	*Cdh5-Cre*	Berry and Rodeheffer, 2013
	White adipocytes	*PdgfRα-Cre*	Berry and Rodeheffer, 2013
	White, inguinal white, and brown adipose tissue	*Retn-Cre*	Mullican et al., 2013

The goal of such systems is to recapitulate the human condition, which can only be done in part. Indeed, mouse models are simply that—models that will never completely recapitulate human PDAC. It is critical to generate these models in a clean background strain to eliminate the potential causative role that genetic variability among chimerics may play when comparing test and control animals, particularly as the complexity of these models increases. The layering of multiple schemes lends itself to amplifying the anomalies produced by one model and potentially augmenting those in another system as they are combined. Despite these caveats, current and future inducible and/or conditional models will lead to a more faithful representation of human disease, which is essential to teasing out the phenotypic and mechanistic aspects of pancreatic cancer that will ultimately improve outcomes in the clinic.

### Conflict of interest statement

The Associate Editor Mouad Edderkaoui declares that, despite having collaborated with author Paul Grippo, the review process was handled objectively and no conflict of interest exists. The authors declare that the research was conducted in the absence of any commercial or financial relationships that could be construed as a potential conflict of interest.
